# Mental health in challenging times: Psychological perspectives for practitioners and society

**DOI:** 10.1192/j.eurpsy.2024.41

**Published:** 2024-08-27

**Authors:** C. Steinebach

**Affiliations:** EFPA, Brussels, Belgium

## Abstract

**Abstract:**

In general, resilience is a process in which the interplay of risk and protective factors of the system itself and its environment is balanced in such a way that positive development opportunities open up. The resilience of a person, a system and a profession is therefore reflected in the ability to shape conditions in such a way that positive coping with challenges and crises is possible as a basis for positive further development. The time of the pandemic and the war in Ukraine has led to a large number of adjustments to psychology as a science, as a profession and as a perspective on life. This is associated with opportunities for positive further development of the discipline. European psychology has so far mastered the challenge of the pandemic and the war in Ukraine very well. The task now is to harness its successes as a multifunctional hub for other sciences, professions and society as a whole. The aim is to develop an identity that strengthens the unity of psychology in its diversity. With wisdom and resilience, psychology is also increasingly facing up to the challenges expressed in the United Nations Sustainable Development Goals (UN SDGs). In the discussion of social and professional change, the possibilities for a joint positive development of all professions in these stressful times become clear.

**Disclosure of Interest:**

None Declared

